# Distinct brain activity patterns associated with traditional Chinese medicine syndromes: a task-fMRI study of mild cognitive impairment

**DOI:** 10.3389/fnins.2025.1555365

**Published:** 2025-03-12

**Authors:** Zhaoying Li, Shanyu Liu, Yuling Shen, Huan Zhao, Zhenwei Chen, Rui Tan, Zhuoling Li, Ling Quan, Dongdong Yang, Min Shi

**Affiliations:** ^1^Department of Neurology, Hospital of Chengdu University of Traditional Chinese Medicine, Chengdu, China; ^2^Department of Integrated Traditional Chinese and Western Medicine, West China Hospital, Sichuan University, Chengdu, China; ^3^Department of Neurology, Shenzhen Hospital of Guangzhou University of Chinese Medicine, Shenzhen, China

**Keywords:** mild cognitive impairment, task-based fMRI, episodic memory, traditional Chinese medicine syndrome, brain activity

## Abstract

**Background:**

Abnormalities in brain activity patterns during episodic memory tasks have been inconsistently reported in amnestic mild cognitive impairment (aMCI) patients using functional magnetic resonance imaging (fMRI). This study applied traditional Chinese medicine (TCM) syndrome differentiation to categorize aMCI patients into distinct subgroups, aiming to clarify the neural mechanisms underlying their cognitive profiles.

**Methods:**

Participants included aMCI patients categorized into the turbid phlegm clouding the orifices (PCO) or spleen-kidney deficiency (SKD) syndrome subgroups, alongside cognitively normal controls (NC) matched for age and gender. Neuropsychological assessments were performed, and fMRI scans were acquired during an episodic memory task involving the recognition of new and old vocabulary. Brain activity across different stages of episodic memory was analyzed using SPM12 and DPABI 7.0 software.

**Results:**

A total of 57 aMCI patients (34 with SKD and 23 with PCO) and 54 healthy controls were involved in the final task-based fMRI analysis. Compared with the NC group, the PCO group exhibited increased brain activation during both encoding and retrieval phases, primarily involving the prefrontal cortex and occipital lobe. Compared with the SKD group, the PCO group demonstrated the elevated activation in the right central sulcus and right insula during the encoding phase. Correlation analysis indicated a specific association between PCO symptom scores and insula activation. No statistically significant differences were found between the SKD and NC groups.

**Conclusion:**

Distinct patterns of fMRI brain activity found in aMCI patients with PCO and SKD syndromes during episodic memory tasks suggest differing neural mechanisms that may contribute to the clinical heterogeneity of aMCI.

## 1 Introduction

Amnestic mild cognitive impairment (aMCI), characterized by impaired episodic memory, is considered as the prodromal stage of Alzheimer’s disease (AD). The annual conversion rate of aMCI to AD ranges from 11.0 to 16.5%, which is 10 times higher than that found in the general elderly population (Oltra-Cucarella et al., 2018). The 2024 guidelines published by the National Institute on Aging and Alzheimer’s Association further emphasise the dynamic and continuous progression of the AD spectrum (Jack et al., 2024). As a critical transitional stage, early identification of aMCI symptoms and imaging biomarkers is essential for developing timely intervention strategies. However, a persistent challenge lies in the considerable clinical heterogeneity of aMCI, as it represents a syndrome with diverse underlying pathophysiological mechanisms rather than a single, uniform disease entity. Patients with aMCI may exhibit divergent cognitive profiles, neuropsychiatric symptoms, treatment responses, and even functional magnetic resonance imaging (fMRI) patterns (Bozoki et al., 2001; Du et al., 2022; Kim et al., 2023; Ma, 2020; Russ and Morling, 2012). For instance, both hyperactivation and hypoactivation of the medial temporal lobe and hippocampus have been reported in MCI patients during memory tasks (Hanseeuw et al., 2011; Heun et al., 2007; Kircher et al., 2007; Petrella et al., 2006).These disparities likely reflects distinct neuropathological trajectories, highlighting the need for novel approaches to delineate clinical subgroups.

Notably, TCM syndrome differentiation provides a unique framework for capturing these variations. TCM syndromes are clinical subtypes identified by TCM practitioners through the systematic observation, summarization, and categorization of the symptoms and signs of aMCI over extended periods. According to the *Guiding Principles for Clinical Research of New Chinese Medicines* (2002 version) and Tian’s findings, the two most prevalant TCM syndromes in aMCI are turbid phlegm clouding the orifices (PCO) and spleen-kidney insufficiency (SKD) syndromes (Miao et al., 2009; Zheng, 2002). SKD syndrome is characterized by clinical manifestations, such as forgetfulness, slow movement, pain, and weakness in the lower back and legs, loose stools, nocturia, hypogonadism, and a weak pulse. PCO syndrome is associated with diminished intelligence, emotional apathy, fatness, somnolence, snoring, phlegm in the throat, and a greasy tongue coating. In a Chinese aMCI cohort, the PCO group demonstrated a robust correlation with situational memory and an elevated risk of progression to AD (Fang et al., 2011). In contrast, SKD syndrome was associated with impairments in language, visuospatial function, and attention (Wang, 2021).

Crucially, these TCM syndrome subgroups may serve as phenotypically anchored “bridges” between clinical heterogeneity and neurobiological mechanisms—a hypothesis increasingly supported by fMRI findings. Task-based fMRI’s high spatiotemporal resolution uniquely enables detection of TCM syndrome-specific neural circuit dysfunctions during memory tasks. For instance, an fMRI study on depression revealed the enhanced functional connectivity in the inferior parietal lobe for patients in the TCM yang-type subgroup, while decreased connectivity was identified in the yin-type subgroup along with more severe cognitive impairment (Xu et al., 2018). Another study identified the lateralization pattern in the bilateral frontal gyrus, distinguishing between deficiency and excess patterns in subcortical vascular mild cognitive impairment (Wang et al., 2022). It can be thus hypothesized that aMCI patients with different TCM syndromes may have different brain activation patterns during episodic memory tasks.

The objective of this study was to examine the characteristics of brain activity patterns in aMCI patients with PCO and SKD syndrome through task-based fMRI. By applying task-based fMRI to an aMCI cohort categorized by TCM subtypes, this study addressed the heterogeneity challenge through a dual approach, combining insights from TCM syndrome classification with modern neuroimaging techniques.

## 2 Methods

### 2.1 Participants

In this study, aMCI subjects who were admitted to the Hospital of Chengdu University of TCM (Chengdu, China) between November 2022 and April 2024 were recruited, along with age- and sex-matched healthy controls from the health check-up center and local community. In accordance with the findings of a preceding study on episodic memory task-based fMRI of MCI (Kircher et al., 2007), we caculated a minimum sample size of 22 with an anticipated power was set at 0.8. Informed consent was obtained from all participants prior to their involvement in the study. The study was approved by the Medical Ethics Committee of the Hospital of Chengdu University of TCM (Approval No. 2022KL-042-02). The inclusion criteria were as follows: (1) Age between 50 and 80 years; (2) Right-handedness; (3) Sufficient audiovisual and comprehension abilities to complete neuropsychological assessments; (4) Diagnosis of aMCI based on the criteria proposed by Petersen et al. (1999) and the Chinese Expert Group on Dementia and Cognitive Impairment (Writing Group of the Chinese Dementia and Cognitive Impairment Diagnosis and Treatment Guidelines, 2018), including memory decline lasting more than 6 months, a global clinical dementia rating (CDR) score of 0.5, and impaired long-term memory, which was defined as a score of at least one standard deviation below the age-adjusted normative mean on the Auditory Verbal Learning Test-HuaShan version (AVLT-H) (Bondi et al., 2014; Zhou et al., 2024); (5) Diagnosis of PCO or SKD syndrome based on TCM Syndrome Score Scale (TCMSSS) in the Chinese guidelines for clinical studies on aMCI (Tian et al., 2008; Miao et al., 2009). The TCMSSS contains specific TCM symptoms, such as “Curdy Fur,” each assigned a corresponding score (Supplementary Table S.1). The syndrome is diagnosed when the total score exceeds 7. Additionally, a higher TCMSSS score indicates more severe TCM symptoms (Tian et al., 2008). In addition, healthy participants underwent a neuropsychological assessment to confirm normal cognition.

The exclusion criteria were as follows: (1) Comorbid conditions, including tumors, severe cardiac, hepatic, renal, or hematological disorders, psychiatric disorders, and medical conditions associated with cognitive impairment (e.g., cerebrovascular disease, Parkinson’s syndrome, normal pressure hydrocephalus, vitamin B1 or B12 deficiency, thyroid dysfunction, alcoholism, carbon monoxide poisoning); (2) Severe visual or auditory impairments; (3) History of drug or alcohol abuse; (4) Contraindications for MRI, such as presence of pacemakers or metal implants, claustrophobia; (5) Hamilton Depression Scale score exceeding 17. Neuropsychological assessments included the Montreal Cognitive Assessment (MoCA), AVLT-H, Boston Naming test, Animal Fluency test, Hamilton Depression Scale, and Hamilton Anxiety Scale. Thresholds for neuropsychological scales are presented in the Supplementary Table S.2.

### 2.2 Memory task presentation

The vocabulary memory task for discriminating old and new words was developed using an event-related design and compiled with E-Prime 3.0 (Psychology Software Tools, Inc., Pittsburgh, USA). The task comprised three cycles, each lasting 224 s. Each cycle included two vocabulary encoding blocks alternating with two vocabulary retrieval blocks. Each encoding block contained 15 words, while each retrieval block presented 30 words, consisting of 15 previously encoded words and 15 novel ones. Words were displayed for 1 s each, with inter-word intervals varying between 2 and 8 s. Participants were instructed to memorize as many words as possible during the encoding phase and to determine whether the words presented during the retrieval phase were old or new. Participants received a detailed briefing on the examination procedure 30 min before the scan and completed training on a laptop using practice tasks different from the formal trial.

A magnet-compatible brain function visual stimulation system (SA-9939; Sinorad Medical Electronics Co., Ltd., Shenzhen, China) was employed to present vocabulary word stimuli. The system comprised a master console, a 40-inch LED display, reflective mirrors, MRI-specific vision calibration lenses, and a two-handed independent response control button box. Participants wore MRI-specific vision calibration lenses to achieve a visual acuity of 4.8 or better. During scanning, words were viewed through a mirror mounted on the head coil, and participants responded by pressing the “yes” or “no” buttons on the response control box. Behavioral data collected during the task were recorded for analysis.

### 2.3 fMRI procedure

Imaging was carried out using a 3.0-tesla GE MRI scanner (DISCOVERY MR750, GE, Berlin, Germany). T1-weighted magnetization-prepared rapid gradient echo structural images were acquired with high-resolution parameters optimized for anatomical precision: repetition time (TR) = 8.208 ms, echo time (TE) = 3.22 ms, field of view (FOV) = 240 × 240 mm^2^, acquisition matrix = 256 × 256, isotropic voxel dimensions = 0.938 × 0.938 × 0.5 mm^3^, flip angle = 7°, slice thickness = 1.0 mm with an interslice gap of 0.5 mm, and a total of 312 slices. Subsequent fMRI acquisitions were performed on all participants by an experienced radiologist utilizing the identical MRI system to ensure standardization of imaging conditions. Whole-brain functional imaging data were captured using an echo-planar imaging sequence designed to maximize temporal and spatial resolution. The protocol included 39 contiguous axial slices with the following specifications: TR = 2,000 ms, TE = 30 ms, FOV = 240 × 240 mm^2^, acquisition matrix = 64 × 64, voxel dimensions = 3.75 × 3.75 × 3.5 mm^3^, slice thickness = 3.5 mm with no interslice gap, and a flip angle of 90°.

### 2.4 Data analysis

The initial 10 volumes of the task-based fMRI data were discarded to ensure signal stabilization, followed by preprocessing using SPM12 7,771 software. Preprocessing steps included slice timing correction and head motion correction for the BOLD images. The middle layer was selected as the reference layer to maintain a consistent scan time across layers. All images were aligned, and head-motion parameter files were generated for correction in subsequent data analysis. T1-weighted structural images were segmented into gray matter, white matter, and cerebrospinal fluid. The segmented gray matter images were thereafter co-registered with the mean functional image. Subsequently, BOLD images were resampled to a resolution of 3 × 3 × 3 mm^3^ and normalized to the Montreal Neurological Institute template using the co-registered gray matter images. Finally, the BOLD data were smoothed using a Gaussian kernel with a 6 mm full-width at half maximum. Images with noticeable blurring, artifacts, or head motion exceeding 3 mm were excluded.

Individual-level brain activation maps were generated using a general linear model in SPM12. Stimulus conditions were categorized as follows: encoding, retrieval-old, retrieval-new, encoding minus retrieval-old, and encoding minus retrieval-new. The individual-level results were thereafter utilized for second-level analysis. Second-level analyses were conducted to identify activated brain regions in groups using one-sample t-test. A false discovery rate (FDR) correction was applied, with the brain mask dimensions set to 61 × 73 × 61, a significance threshold of *p* < 0.05, and a cluster size of ≥20 voxels. The FDR correction has superior sensitivity while controlling the false positive rate, facilitating the detection of differences in brain region activation (Genovese et al., 2002). The thresholds for corrected *p*-value and cluster size were considered to select relatively sensitive boundaries for the exploratory analyses of the TCM subgroups in this study, with reference to previous studies of a similar nature (Hanseeuw et al., 2011; Kircher et al., 2007; Petrella et al., 2006; Trivedi et al., 2008). Group image comparisons were conducted using DPABI 7.0 software, employing two-sample t-test with age, gender, and education as covariates. Additionally, Gaussian random field correction was applied, with a cluster-level threshold of *p* < 0.05 (two-tailed). Brain activity results were reported according to the AAL3 template (Rolls et al., 2020).

Clinical data were statistically analyzed using SPSS 26.0 software (IBM Corporation, Armonk, NY, USA). Continuous variables were expressed as mean (standard deviation), and two-sample *t*-test was employed for making comparison between groups. Categorical variables were expressed as frequency (percentage) and subjected to comparison using Chi-square test. Count data, such as scale scores, were expressed as median (interquartile range) and compared using the Mann–Whitney U test. Missing data were addressed through multiple imputation prior to analysis. Pearson correlation analysis was carried out between clinical data and brain activation maps, with age, gender, and education included as covariates. *p* < 0.005 was considered statistically significant.

## 3 Results

### 3.1 General characteristics

A total of 68 MCI subjects (38 with SKD and 30 with PCO) and 60 healthy controls participated in the study and completed the neuropsychological assessments. Notably, 5 subjects voluntarily withdrew from the study before or during the scanning. Moreover, 7 cases were excluded due to substandard fMRI images and 5 due to failure to complete the stimuli task. Ultimately, task-based fMRI data from 57 cases of MCI (34 with SKD and 23 with PCO) and 54 healthy controls were included in the analyses. The results of the comparative analysis of general characteristics between the MCI and NC groups are presented in Table 1. The PCO and SKD groups demonstrated no statistically significant differences in general characteristics and neuropsychological assessments (*p* > 0.05) (Supplementary Table S.3).

**Table 1:** Participants’ general characteristics.

Items	MCI group (*n* = 68)	NC group (*n* = 60)	*p*
Age (years)^*^, mean (SD)	59.9 (7.4)	59.7 (6.52)	0.872
Gender^**^, Female, *n* (%)	49 (72.1%)	49 (81.7%)	0.200
Education (years)	9 (7,12)	12 (9,15)	0.001
MoCA scores	20 (18,22)	27 (25,28.75)	<0.001
AVLT (instant)	13 (11,15)	20 (17,22)	<0.001
AVLT (short-term)	4 (3,5)	7 (6,9)	<0.001
AVLT (long-term)	3 (2,4)	7.5 (6.5,9)	<0.001
AVLT (cued recall)	3 (1,4)	7 (7,9)	<0.001
AVLT (recognition)	19 (17,20)	23 (22,24)	<0.001
Hamilton Depression Scale	8 (5,11.5)	4 (3,6)	<0.001
Hamilton Anxiety Scale	8 (5.5,12)	6 (3,9)	0.001
Boston naming test	17.5 (22,24)	22 (25,27)	<0.001
Animal fluency test	12 (14,16)	15 (16,19)	<0.001
Episodic memory performance during scanning			
	(*n* = 57)	(*n* = 54)	
Overall accuracy (%)	64 (54,67)	67.5 (64,72)	0.005
Accuracy_ old (%)	63 (54,71.5)	67.5 (64,72)	0.344
Accuracy _new (%)	59 (52,67.5)	69 (61,76)	0.009

The remaining variables were expressed as median (interquartile range) and compared using the Mann–Whitney U test. MCI, mild cognitive impairment, NC, normal control, SD, standard deviation, MoCA, Montreal cognitive assessment, AVLT, auditory verbal learning test.

* Two-sample *t*-test.

** Chi-square test.

### 3.2 Task-based fMRI results in the PCO and SKD groups

#### 3.2.1 Comparison with the NC group

**Figure 1:**
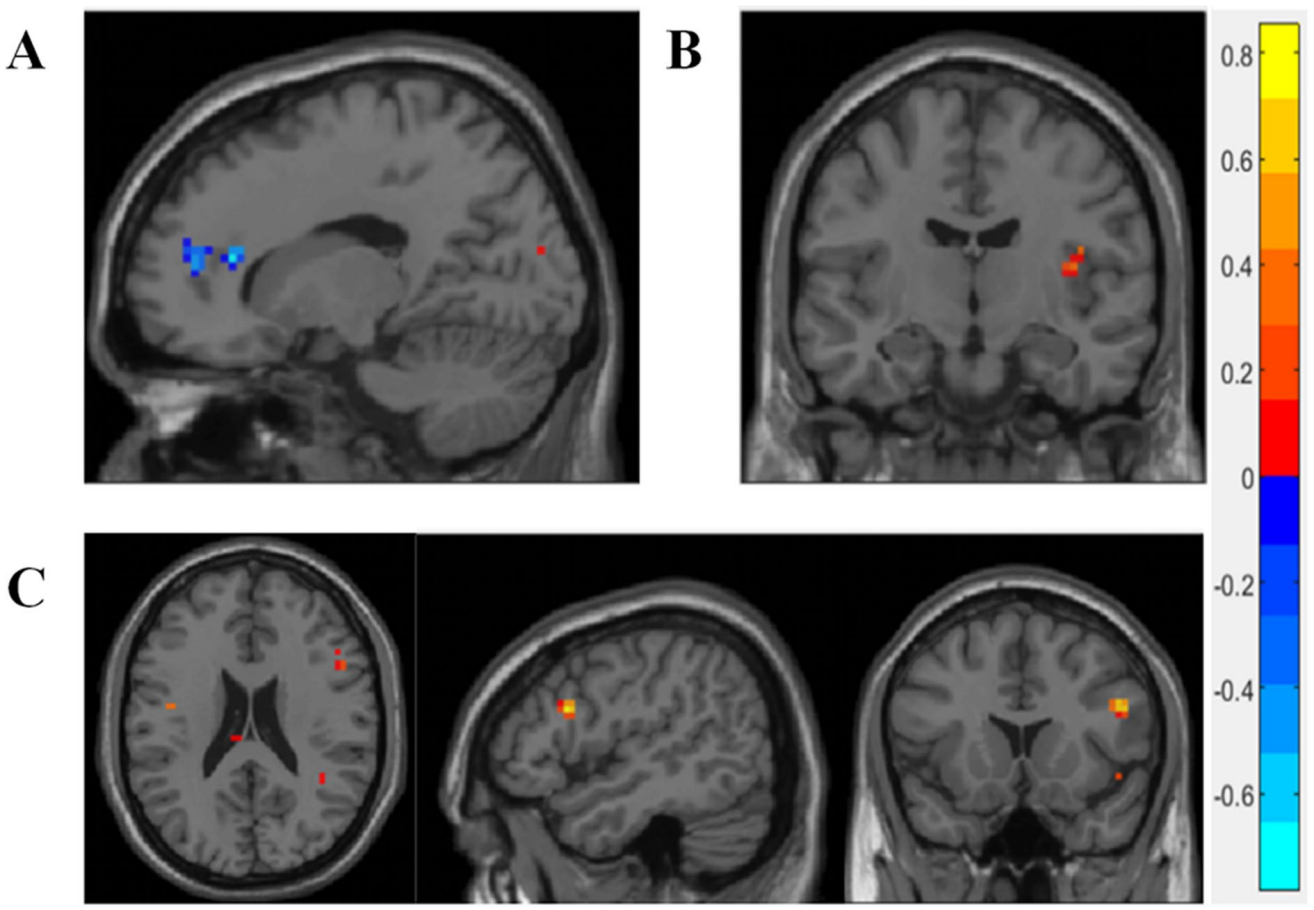
The brain activity of significant differences between groups. **(A)** PCO vs. NC. **(B)** PCO vs. SKD. **(C)** MCI vs. NC. Blue color represents increased activation in the PCO group or MCI group and red color indicates decreased activation. Two-sample *t*-tests was used, with age, gender, and education as covariates. Gaussian Random Field GRF correction was applied cluster *p* value<0.05, two-tailed was set. PCO: Turbid phlegm clouding the orifices syndrome subgroup; SKD: Spleen-kidney insufficiency syndrome subgroup; MCI: Mild cognitive impairment group; NC: Normal control group.

Compared with the NC group, the PCO group demonstrated augmented brain activity in the right middle occipital gyrus during the encoding phase (Figure 1A). Additionally, during the retrieval-old phase, the PCO group exhibited enhanced activation in the left dorsolateral superior frontal gyrus and the right superior occipital gyrus, alongside reduced activity in the left superior parietal gyrus (corrected *p* < 0.05) (Figure 1A). During the retrieval-new phase, elevated activation was found in the left dorsolateral superior frontal gyrus and the left cuneus (corrected *p* < 0.05) (Figure 1A). The specific brain regions exhibiting differential activation are detailed in Supplementary Table S.4. No statistically significant differences in brain activation were identified between the SKD and NC groups (corrected *p* > 0.05).

#### 3.2.2 Comparison between the PCO and SKD groups

The PCO group exhibited elevated activation in the right central sulcus area and right insula during the encoding phase compared with the SKD group (corrected *p* < 0.05) (Figure 1B, Supplementary Table S.5). However, no significant difference was identified between the groups during the entire retrieval phase or the encoding-minus-retrieval phase (corrected *p* > 0.05).

#### 3.2.3 Correlation between brain activity and TCMSSS

**Figure 2:**
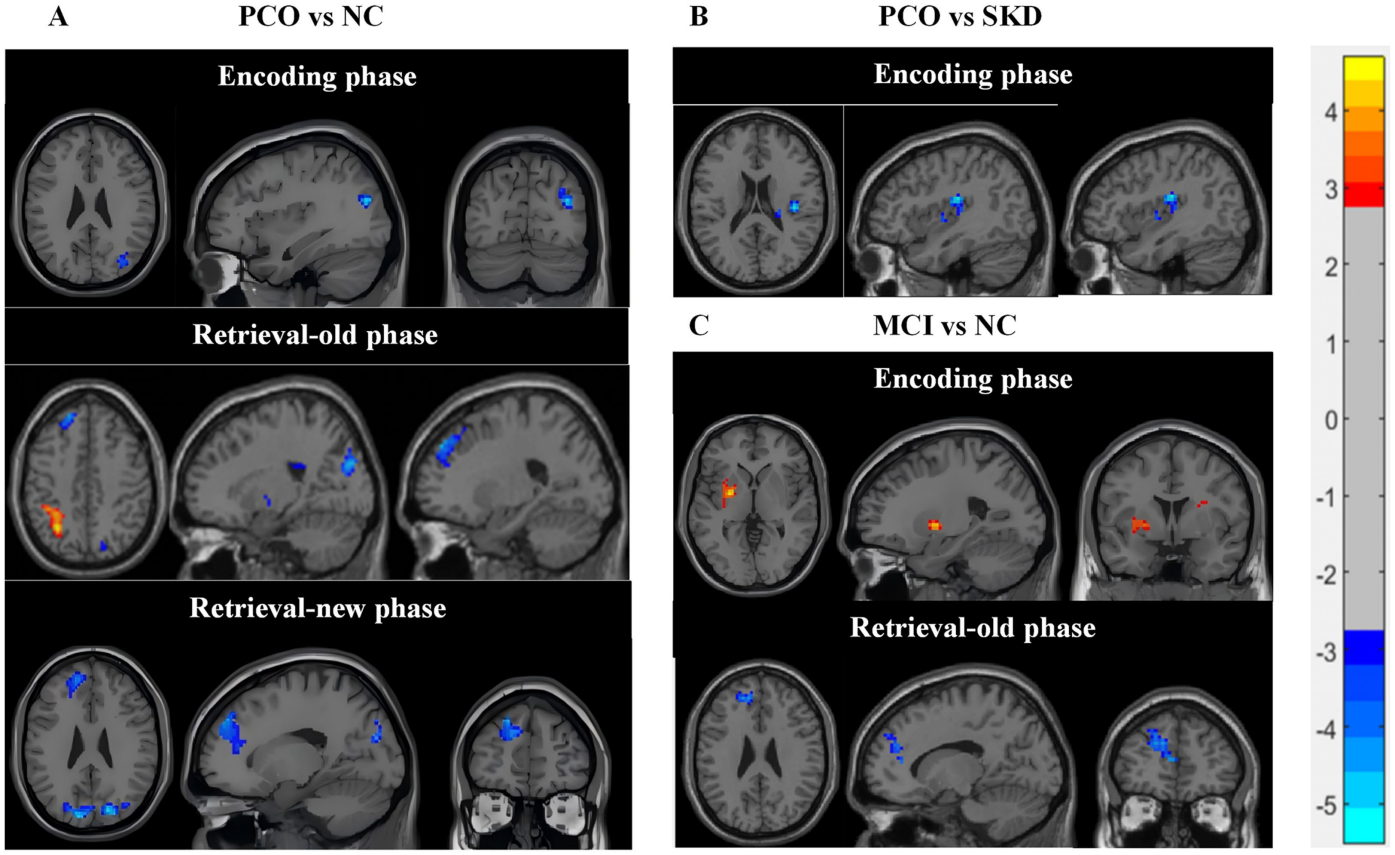
Correlation between brain activity and TCMSSS. **(A,B)** During the encoding phase, the TCM evidence score of PCO syndrome was positively correlated with the right insula, while negatively correlated with the anterior cingulate and paracingulate gyri. **(C)** The TCM evidence score of SKD syndrome was positively correlated with the right triangular part of inferior frontal gyrus during the encoding phase. Blue color represents negative correlation, while red color represents positive correlation. Pearson correlation analysis was carried out, with age, gender, and education included as covariates. *p* < 0.005 was considered statistically significant.

The TCMSSS is a quantitative measure, indicating the degree of conformity between an individual and a specific TCM syndrome. Higher scores indicate a greater likelihood of diagnosis with the corresponding TCM syndrome. During the encoding phase, the TCMSSS of PCO syndrome was positively correlated with the right insula, while negatively correlated with the anterior cingulate and paracingulate gyri (*p* < 0.005) (Figures 2A,B). For SKD syndrome, the TCMSSS was positively correlated with activation in the right triangular part of the inferior frontal gyrus (*p* < 0.005) (Figure 2C). No significant correlations were identified during the retrieval phase.

### 3.3 Comparison of all MCI patients and the NC group

#### 3.3.1 Encoding phase

During the encoding phase, both MCI and NC groups exhibited activation in several brain networks, such as central executive network, dorsal attentional network (DAN), and visual network, as well as negative activation in the DMN (Supplementary Figure S.1, Supplementary Table S.6). Compared with the NC group, the MCI group demonstrated diminished activation in the left putamen and right insula (corrected *p* < 0.05) (Figure 1C, Supplementary Table S.7).

#### 3.3.2 Retrieval phase

Throughout the retrieval phase, both the MCI and NC groups exhibited widespread brain region activation and negative activation of the DMN (Supplementary Figure S.1, Supplementary Table S.8). The MCI group showed enhanced activation in the prefrontal cortex during the retrieval-old phase, including left dorsolateral superior frontal gyrus, medial superior frontal gyrus, and left anterior cingulate cortex (corrected *p* < 0.05) (Figure 1C, Supplementary Table S.7).

### 3.4 Correlation analysis between cognitive performance and brain activity

**Figure 3:**
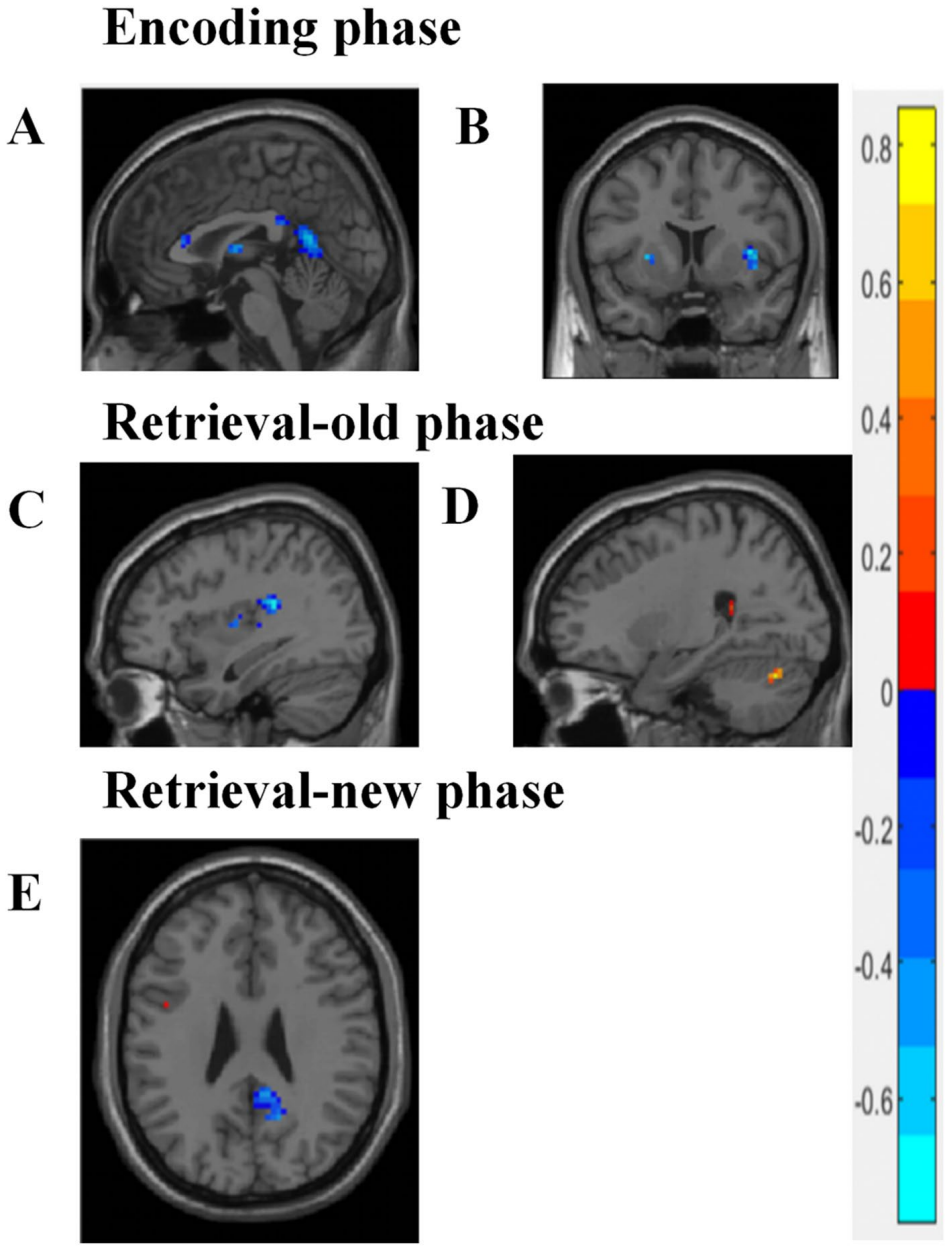
Correlation analysis between cognitive performance and brain activity. **(A,B)** During the encoding phase, a negative correlation was identified between the left precuneus and AVLT instant scores, while a negative correlation was found between the right insula and MoCA scores (*p* < 0.005). **(C,D)** During the retrieval-old phase, the right superior cerebellum activity was found to be positively correlated with AVLT recognition scores, whereas the accuracy of old and new vocabulary recognition was negatively correlated with the right insula (*p* < 0.005). **(E)** During the retrieval-new phase, the accuracy of old and new vocabulary recognition was negatively correlated with the posterior cingulate gyrus (*p* < 0.005). Blue color represents negative correlation, while red color indicates positive correlation. Pearson correlation analysis was carried out, with age, gender, and education included as covariates. *p* < 0.005 was considered statistically significant.

The correlations between cognitive performance and brain region activation were examined. During the encoding phase, a negative correlation was found between the left precuneus and AVLT instant scores, while a negative correlation between the right insula and MoCA scores was also identified (*p* < 0.005) (Figures 3A, B). In the retrieval-old phase, activity in the right superior cerebellum was positively correlated with AVLT recognition scores, and the accuracy of old and new vocabulary recognition was negatively correlated with the right insula (*p* < 0.005) (Figures 3C,D). In the retrieval-new phase, the accuracy of old and new vocabulary recognition was negatively correlated with the posterior cingulate gyrus (*p* < 0.005) (Figure 3E).

## 4 Discussion

Chinese medicine represents a significant component of the global healthcare system and has been incorporated into the International Classification of Diseases, 11th Revision (Lam et al., 2019). Integrating TCM syndrome diagnosis with investigations into its biological underpinnings provides deeper insights into disease heterogeneity. The present task-based fMRI findings indicate that PCO and SKD in aMCI are not only reflected in TCM symptomatology, but are also associated with distinct brain activation patterns during episodic memory processing.

Distinct patterns of brain activity were predominantly identified in the PCO syndrome group. During the encoding phase, the PCO group exhibited the elevated activation in the middle occipital gyrus compared with the NC group. In the extraction phase, the PCO group continued to demonstrate hyperactivation, predominantly in the dorsolateral prefrontal cortex (DLPFC) and occipital lobes. Increased activation in the occipital lobe has also been reported in previous studies on MCI, suggesting compensatory enhancement of visual input processing (Kircher et al., 2007). In particular, we offered visual stimuli during scanning. The DLPFC, a key region involved in attention and executive function, was notably affected by AD pathology (Joseph et al., 2021). The findings suggested that the PCO group encountered cognitive challenges during retrieval, necessitating the active engagement of recall strategies. Previous research has demonstrated a significant association between SKD and impairments in visuospatial ability and executive function (Lin et al., 2022; Wang, 2021). Therefore, tasks, such as pointer position discrimination, stroop, and n-back may be more effective for investigating the neuroimaging mechanisms of SKD. Additionally, a significant correlation was identified between the right triangular part of the inferior frontal gyrus and the TCMSSS score for SKD. A previous study demonstrated the importance of the right inferior frontal gyrus in response strategies for complex behaviors (Dippel and Beste, 2015). Therefore, a multi-task sequence based on the stop-change paradigm is also worthy of consideration.

The right insula, a key brain region of interest in this study, exhibited significant differences between the PCO and SKD groups. The insula plays a crucial role in integrating internal and external sensory information and is pivotal for allocating and regulating attention, thereby influencing learning and memory processes (Menon and Uddin, 2010). The right anterior insula has been reported to coordinate the activation of the central executive network and the deactivation of the DMN during functional tasks (Ulrich et al., 2022). In aMCI and AD, disrupted positive functional connectivity of the insula across networks has been linked to deficits in episodic memory (Liu et al., 2018; Xie et al., 2012). Previous studies have reported conflicting findings regarding insular activation during memory encoding in aMCI, involving both hyperactivation and activation deficits (Gu and Zhang, 2019; Machulda et al., 2009). The heterogeneity of clinical samples may be a key factor contributing to these inconsistencies. In certain aMCI populations, such as the PCO group in this study, insular hyperactivation may serve as a compensatory mechanism for disrupted cognitive network connectivity. Furthermore, the insula is involved in emotion perception and regulation (Zhang et al., 2022). Notably, the TCMSSS score for PCO syndrome was positively correlated with right insular activation. In contrast, the SKD group did not exhibit insular activation for effective cognitive compensation. These findings suggested that the two TCM subgroups differed in both their strategies and capacities for mobilizing cognitive resources. Additionally, emotional state could be a contributing factor. During the retrieval phase, no significant differences in brain activity were identified between the two subgroups, indicating the possibility of a shared pathological mechanism.

In conclusion, this task-based fMRI study provided valuable insights into the investigation of TCM syndromes in aMCI. Additionally, other MRI techniques have also been explored in previous research. For instance, Yang (2012) employed magnetic resonance spectroscopic imaging in vascular cognitive impairment and found that hippocampal and angular gyrus inositol reduction was characteristic of deficiency syndrome, whereas increased lactate level in the posterior cingulate gyrus was indicative of excess syndrome. From a pathological perspective, cerebrospinal fluid amyloid β-protein-42 and tau protein levels were reported to be higher in aMCI patients with PCO syndrome relative to those with SKD syndrome, while the SKD subgroup exhibited a remarkable deficiency in acetylcholine (Li et al., 2017). These findings align with TCM perspective on the concept of PCO and SKD syndromes. The PCO syndrome corresponds to an excess condition, indicating the accumulation of pathological products, whereas the SKD syndrome reflects a deficiency state associated with systemic dysfunction. A resting-state fMRI study by Wang et al. (2022) found the increased spontaneous brain activity in the left superior temporal gyrus, right middle temporal gyrus, and brainstem in subcortical vascular MCI with a deficiency pattern compared with those with an excess pattern. In contrast to Wang et al.’s findings, the present study identified hyperactivation features in the PCO syndrome compared with both the normal and SKD groups. Notably, TCM subgroups exhibit different brain activity features between the task state and resting states. In addition, discrepancies exist in the mechanisms of cognitive impairment between subcortical vascular cognitive impairment and aMCI.

The present study replicated the mechanism of situational memory impairment through fMRI in aMCI, as evidenced by previous studies (Clément and Belleville, 2010; Li et al., 2015; Li et al., 2013). The processes of episodic memory formation and retrieval rely on dynamic interactions among multiple cognitive networks. Previous studies have indicated that during task states, the DMN should exhibit deactivation, while the DAN becomes activated (Smallwood et al., 2021). The frontoparietal control network (FPN), involving regions, such as the lateral prefrontal cortex, anterior cingulate cortex, and inferior temporal lobe, is critical for mediating the flexible interaction between the DMN and the DAN. This network facilitates the execution of specific cognitive tasks and decision-making processes (Nee, 2021; Spreng et al., 2010). During the encoding phase, all participants displayed characteristic patterns of DMN deactivation, DAN activation, and FPN engagement. However, the extent of both activation and deactivation was notably reduced in the MCI group relative to the NC group. Thus, cognitive impairment in aMCI may result from imbalances across multiple cognitive network regions. This was also supported by the reduced coordination and diminished activation of the insula and functional shell nuclei in aMCI patients. Both the insula and shell nucleus play a fundamental role in transmitting and integrating brain information across different cognitive networks (Zhang et al., 2024; Mandzia et al., 2009). The results of the retrieval phase demonstrated an increase in both the intensity and extent of activated clusters relative to the encoding phase. The activation extended primarily to subcortical nuclei, including the thalamus, basal ganglia, cerebellum, and brainstem. Negative activation was also identified in regions of the DMN. In contrast to the encoding phase, the retrieval phase involved the recognition of old words and the introduction of new words, engaging cognitive domains, such as attention, vision, perception, word recognition, semantic memory, and situational memory (Ward, 2018). Consequently, the retrieval phase necessitates the involvement of a broader range of cognitive processes, requiring the activation of additional reserve resources beyond those employed during encoding. During the retrieval phase, patients with MCI demonstrated increased cortical activation in the left dorsolateral superior frontal gyrus, medial superior frontal gyrus, and left anterior cingulate. The hyperactivation of FPN regions aligns with findings of previous studies (Heun et al., 2007; Remy et al., 2005; Trivedi et al., 2008), suggesting a compensatory mechanism for cognitive resource allocation.

## 5 Limitations

This study had several inherent limitations. The subjects in our study were younger, predominantly female, and had less severe cognitive impairment, which may have diminished the representativeness of the sample. Therefore, the development of more precise clinical stratification strategies, incorporating a wider range of TCM evidence types, is essential for future research. Given the clinical heterogeneity of aMCI, the differences in symptoms and treatment response across different TCM syndromes deserve further exploration. Additionally, careful consideration should be given to potential confounding factors, including age, gender, education, comorbidities, and emotional state, when interpreting the findings. The cross-sectional design of the study also limits the ability to assess the progression or regression of aMCI. Future research should incorporate a longitudinal follow-up design to enhance understanding of the predictive value of TCM syndromes and distinct brain activation patterns in disease progression.

## 6 Conclusion

This study demonstrated that patients with aMCI exhibit significant impairments in episodic memory alongside aberrant brain activity. Moreover, differences in brain activation patterns between SKD and PCO syndromes were identified, suggesting distinct neural mechanisms underlying the symptoms associated with these TCM syndromes. These findings provide a novel perspective on the neural mechanisms of aMCI and lay a scientific foundation for TCM treatments tailored to syndrome differentiation.

## Data Availability

The raw data supporting the conclusions of this article will be made available by the authors, without undue reservation.
