# Supplementary material for: Distinct brain activity patterns associated with traditional Chinese medicine syndromes: a task-fMRI study of mild cognitive impairment
Source: Front Neurosci. 2025 Mar 12;19:1555365. doi: 10.3389/fnins.2025.1555365 (PMC11937078; doi:10.3389/fnins.2025.1555365)
Supplement: Supplementary file 1 [file Data_Sheet_1.DOCX]

**Supplementary tables and Figures**

| Table S.1 Traditional Chinese medicine evidence score |
| --- |
| Table S.2 Cutoff values for abnormal neuropsychological scale scores |
| Table S.3 Comparison of general characteristics between SKD and PCO syndromes |
| Table S.4 Comparison of brain activation between PCO and NC group |
| Table S.5 Comparison of brain activation between PCO and SKD group |
| Table S.6 Brain activation during encoding phase |
| Table S.7 Brain activation during retrieval phase |
| Table S.8 Comparison of brain activation between MCI and NC group |
| Figure S.1 The brain activity in the MCI and NC groups |

Table S.1. Traditional Chinese medicine evidence score

| Turbid phlegm clouding the orifices syndrome | | Spleen-kidney deficiency syndrome | |
| --- | --- | --- | --- |
| Symptom | Score | Symptom | Score |
| Apathic expression or lack of speech | 2 | Waist pain | 3 |
| Dull expression or unresponsive | 3 | Soreness and weakness of waist and knees | 4 |
| Somnolence | 5 | Backache | 5 |
| Sticky mouth | 2 | Tinnitus like chirping of cicadas | 2 |
| Spiting out or vomiting phlegm | 3 | Deef | 3 |
| A lot of sticky phlegm | 4 | Atrophic or cold auricle | 4 |
| Snoring and sticky mouth | 5 | Loose stools | 2 |
| Nonspecific dizziness | 2 | Urinary incontinence | 3 |
| Fatness | 2 | Urinary and fecal incontinence | 4 |
| Dark complexion | 3 | Cold hands and feet | 1 |
| Slimy tongue fur | 2 | Fear of cold | 2 |
| Sticky and slimy tongue fur | 4 | Loss of hair | 1 |
| Curdy fur | 6 | Loose teeth | 2 |
| Enlarged tongue | 3 | Loss of teeth | 3 |
| Enlarged and teeth-marked tongue | 5 | Incomplete emptying | 2 |
| Slippery pulse | 2 | Frequent nocturia | 3 |
|  |  | Decreased sexual function | 1 |
|  |  | erectile dysfunction | 2 |
|  |  | Red tip of tongue | 1 |
|  |  | Light tongue | 2 |
|  |  | Light and teeth-marked tongue | 3 |
|  |  | Thin and white tongue fur | 1 |
|  |  | Thin, slimy, and white tongue fur | 2 |
|  |  | White and slippery tongue fur | 3 |
|  |  | Weak pulse | 1 |

Table S.2 Cutoff values for abnormal neuropsychological scale scores

| Domain | Neuropsychological scale | Cut-off values for abnormality |
| --- | --- | --- |
| Screening | Montreal cognitive assessment | Cognitive impairment was identified as:  years of education = 0: ≤ 13 points;  years of education ＜ 7 years: ≤ 19 points;  years of education ≥ 7 years: ≤ 24 points. |
| Overall cognitive assessment | Clinical dementia rating | Global score = 0.5: mild cognitive impairment  Global score = 1: dementia (excluded) |
| Memory | Auditory Verbal Learning Test-HuaShan version | Memory impairment was identified as one standard deviation decline in long-term recall scores (50-59 years old ≤ 5 points, 60-69 years old ≤ 4points, and 70-79 years old≤ 3points) and/or recognition scores (50-59 years old ≤ 20 points, 60-69 years old ≤ 19points, and 70-79 years old≤ 18) |
| Language | Boston naming test-30 | Naming impairment was identified as:  junior high school group, ≤ 19 points;  junior high school group, ≤ 21 points;  University group, ≤ 22 points. |
|  | Animal fluency test | Language fluency was identified as:  junior high school group, ≤ 12 points;  junior high school group, ≤ 13 points;  University group, ≤ 14 points. |
| Emotion | Hamilton depression scale | Definitely depression was defined as a score of more than 18 points, and should have been excluded from the study. |
|  | Hamilton anxiety scale | Definitely anxiety was defined as a score of more than 14 points, and should have been excluded from the study. |
| Traditional Chinese Medicine | Traditional Chinese Medicine evidence score | A score greater than or equal to 7 was diagnosed as a specific TCM syndrome. |

Table S.3 Comparison of general characteristics between SKD and PCO syndromes

| Items | SKD subgroup(n = 38) | PCO subgroup(n = 30) | *P* value |
| --- | --- | --- | --- |
| Age (years)*, mean (SD) | 60.11(7.43) | 59.57(7.49) | 0.768 |
| Gender**, Female , n (%) | 30(78.9%) | 19(63.3%) | 0.141 |
| Education (years) | 9(6,12) | 9(7,12) | 0.980 |
| Montreal Cognitive Assessment | 20.5(18,22) | 20(18,21) | 0.464 |
| AVLT (instant) | 13(11,15) | 13(11,15) | 0.916 |
| AVLT(short-term) | 4(3,5) | 4(3,5) | 0.970 |
| AVLT(long-term) | 3(2,4) | 3(3,4) | 0.819 |
| AVLT(cued recall) | 2.5(1,4) | 1(3,4) | 0.612 |
| AVLT(recognization) | 19(16,20) | 18.5(17,20) | 0.741 |
| Hamilton Depression Scale | 8(5,11) | 8(5,12) | 0.598 |
| Hamilton Anxiety Scale, | 8(6,12) | 8(5,13) | 0.638 |
| Boston naming test | 20.5(17,25) | 22(20,24) | 0.527 |
| Animal fluency test | 13(12,15) | 14(12,16) | 0.245 |
| Episodic memory performance during fMRI | | |  |
|  | (n = 34) | (n = 23) |  |
| Overall ACC (%) | 68.5(55.5,64) | 64.5(51,61) | 0.005 |
| ACC_ Old (%) | 71(55.5,65) | 73(51.5,62.5) | 0.344 |
| ACC_New (%) | 69(55,64) | 61(50.5,57) | 0.009 |

*: Independent samples t-test. **:Chi-square test. The remaining variables were expressed using median (interquartile range) and compared with Mann-Whitney test.

AVLT: Auditory verbal learning test, SD: Standard deviation, SKD: Syndrome of spleen-kidney deficiency, PCO: Turbid phlegm clouding the orifices.

Table S.4 Comparison of brain activation between PCO and NC group

| Condition | Encephalic Region | Left/  Right | Volume Pixel | Peak Point | | | |
| --- | --- | --- | --- | --- | --- | --- | --- |
|  |  |  |  | T value | x | y | z |
| Encoding | Middle occipital gyrus | Right | 109 | -5.3276 | 6 | 75 | 4 |
| Retrieval-old | Superior frontal gyrus, dorsolateral | Left | 170 | -5.124 | 18 | 5 | 6 |
|  | Superior occipital gyrus | Right | 179 | -4.6103 | 8 | 81 | 7 |
|  | Superior parietal gyrus | Left | 109 | 4.7112 | 30 | 60 | 5 |
| Retrieval-new | Cuneus | Left | 270 | -5.2458 | 12 | 87 | 7 |
|  | Superior frontal gyrus, dorsolateral | Left | 179 | -4.6103 | 15 | 1 | 0 |
| Encoding minusRetrieval-new | Superior frontal gyrus, medial | Left | 165 | 4.0638 | 9 | 5 | 5 |

Table S.5. Comparison of brain activation between PCO and SKD group

| Condition | Encephalic Region | Left/  Right | Volume Pixel | Peak Point | | | |
| --- | --- | --- | --- | --- | --- | --- | --- |
|  |  |  |  | T value | x | y | z |
| Encoding | Rolandic operculum, Insula | Right | 129 | -4.0986 | 39 | -21 | 21 |

Table S.6 Brain activation during encoding phase

| Encephalic Region | Left/  Right | Volume Pixel | Peak Point | | | |
| --- | --- | --- | --- | --- | --- | --- |
|  |  |  | T score | x | y | z |
| **NC group** |  |  |  |  |  |  |
| Precentral, Frontal_Mid, Frontal_Inf_Triangular,  Insula | Left | 3348 | 10.5629 | -45 | 0 | 48 |
| Occipital_Inf, Fusiform | Right | 484 | 10.3199 | 39 | -69 | -9 |
| Temporal_inf, Fusiform, Occipital_Mid | Left | 261 | 9.2507 | -42 | -54 | -9 |
| Parietal_Sup, Parietal_Inf | Left | 198 | 7.7857 | -30 | -63 | 48 |
| Supplementary motor area | Left | 489 | 7.5449 | -6 | 0 | 63 |
| Temporal pole_Sup | Left | 65 | 4.5881 | -48 | -39 | 18 |
| Lingual, Parahippocampal | Left | 33 | -4.2187 | -24 | -42 | -3 |
| Parahippocampal | Right | 38 | -4.7402 | 27 | -39 | -6 |
| Cingulum_Ant, Frontal_Sup_Medial | Right | 314 | -4.746 | 12 | 45 | 9 |
| Precuneus_Right, Precuneus_Left | Left  and right | 3817 | -7.863 | 3 | -54 | 33 |
| **MCI group** |  |  |  |  |  |  |
| Occipital_Inf/Occipital_Mid | Left | 113 | 7.128 | -24 | -87 | -6 |
| Fusiform/ Occipital_Mid | Right | 93 | 7.5492 | 27 | -84 | -3 |
| SupraMarginal | Left | 20 | 5.7981 | -45 | -39 | 24 |
| Frontal_Inf_Oper, Precentral, Frontal_Inf_  Triangular | Left | 247 | 5.2892 | -39 | 15 | 33 |
| Lingual | Right | 53 | -5.1172 | 15 | -66 | -6 |
| Precuneus | Right | 367 | -6.737 | 18 | -57 | 21 |

Mid: middle, Inf: inferior, Sup: superior, Ant: anterior, Oper: Operculum

Table S.7 Comparison of brain activation between MCI and NC group

| Condition | Encephalic Region | Left/  Right | Volume Pixel | Peak Point | | | |
| --- | --- | --- | --- | --- | --- | --- | --- |
|  |  |  |  | T value | x | y | z |
| Encoding | Putamen | Left | 30 | 4.1641 | -27 | -3 | 0 |
|  | Insula | Right | 31 | 3.68 | 36 | -12 | 24 |
| Retrieval-old | Superior frontal gyrus (dorsolateraland medial), and anterior cingulate cortex | Left | 135 | -3.9951 | -18 | 51 | 36 |

Table S.8 Brain activation during retrieval phase

| Encephalic Region | Left/  Right | Volume Pixel | Peak Point | | | |
| --- | --- | --- | --- | --- | --- | --- |
|  |  |  | T score | x | y | z |
| **NC group** |  |  |  |  |  |  |
| Parietal_Inf, Parietal_Sup, Postcentral, Precentral, Frontal_Mid, Frontal_Inf_triangular part, Frontal_Inf_Oper, Supplementary motor area, Temporal_Mid, Fusiform, Cerebelum, Vermis, Putamen, Caudate, Thalamus, Cerebellum | Left  and right | 14434 | 12.9724 | -36 | -42 | 45 |
| Temporal_Sup | Right | 20 | -4.2244 | 54 | -3 | -9 |
| Lingual | Right | 40 | -4.3429 | 12 | -69 | -3 |
| Cingulum_Ant, Frontal_Sup_Medial | Left  and right | 652 | -6.4574 | 3 | 48 | 12 |
| Cuneus, Precuneus, Occipital_Sup | Left  and right | 1932 | -12.8825 | 18 | -81 | 24 |
| Angular | Left  and right | 228 | -7.2653 | -51 | -60 | 36 |
| Frontal_Sup | Left  and right | 146 | -6.4704 | -24 | 30 | 51 |
| **MCI group** |  |  |  |  |  |  |
| Precentral, Postcentral, Cingulate_Mid, Frontal_Inf_Tri, Frontal_Inf_Oper, Frontal_Mid, Caudate, putamen, Supramarginal, Rolandic_Oper,  Temporal_Sup, Fusiform, Insula, Vermis, Cerebellum, Thalamus | Left  and right | 10195 | 10.8858 | -45 | 3 | 18 |
| Cingulum_Post | Left  and right | 97 | -4.0592 | 6 | -48 | 30 |
| Cingulum_Ant | Right | 40 | -3.7913 | -3 | 39 | 12 |

Mid: middle, Inf: inferior, Sup: superior, Ant: anterior, Oper: Operculum, Post: posterior.


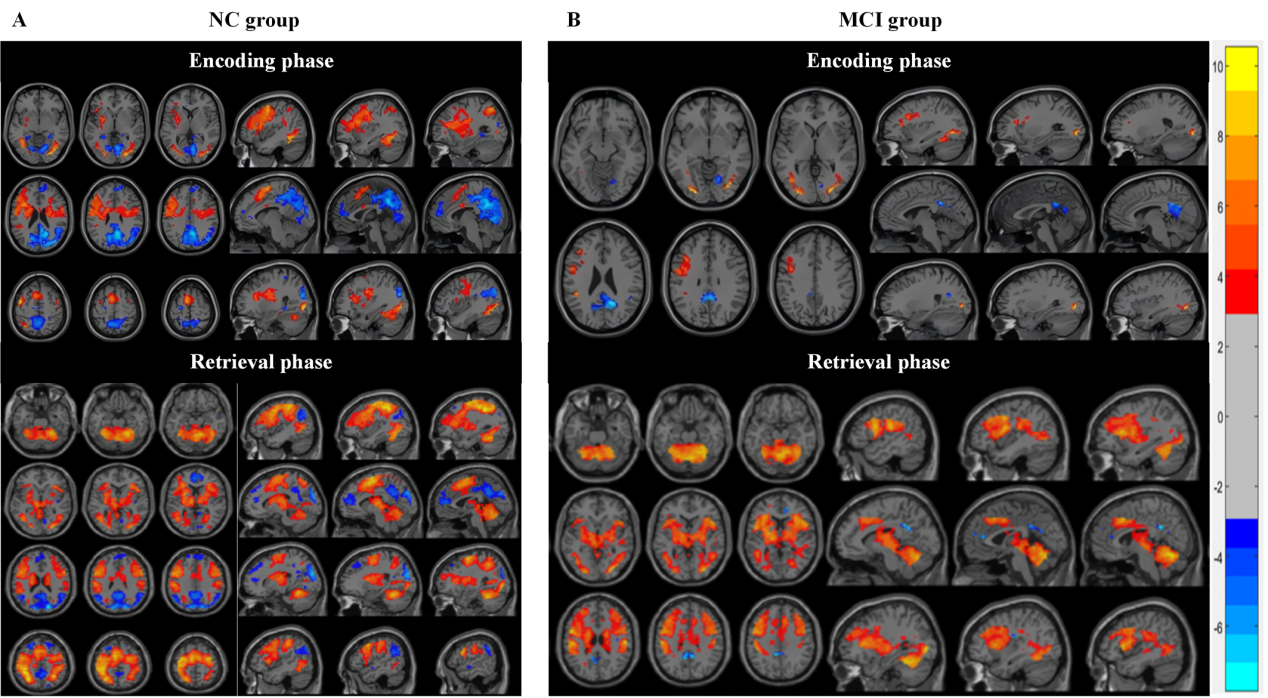


Figure S.1 The brain activity in the MCI and NC groups. (A) NC group. (B) MCI group. Blue color represents negative activation and red color indicates activation. MCI: Mild cognitive impairment group; NC: Normal control group. One-sample T test was used to group-level analysis. A false discovery rate correction was applied, with the brain mask dimensions set to 61 × 73 × 61, a significance threshold of P < 0.05, and a cluster size of ≥20 voxels.
